# Generation and comprehensive analysis of *Synechococcus elongatus*–*Aspergillus nidulans* co-culture system for polyketide production

**DOI:** 10.1186/s13068-023-02283-6

**Published:** 2023-03-01

**Authors:** Jie Feng, Jingwei Li, Dongxia Liu, Yuxian Xin, Jingrong Sun, Wen-Bing Yin, Tingting Li

**Affiliations:** 1 School of Basic Medical Sciences, Lanzhou University, Lanzhou, 730000 China; 2 State Key Laboratory of Mycology, Institute of Microbiology, Chinese Academy of Sciences, Beijing, 100101 People’s Republic of China

**Keywords:** Co-culture, Cyanobacteria, *Aspergillus*, Secondary metabolite, Sucrose, Carbohydrate, Glycogen

## Abstract

**Background:**

Artificial microbial consortia composed of heterotrophic and photoautotrophic organisms represent a unique strategy for converting light energy and carbon dioxide into high-value bioproducts. Currently, the types of desired bioproducts are still limited, and microbial fitness benefit rendered by paired partner generally needs to be intensified. Exploring novel artificial microbial consortia at a laboratory scale is an essential step towards addressing this unmet need. This study aimed to conduct and analyze an artificial consortium composed of cyanobacterium *Synechococcus elongatus* FL130 with the filamentous fungus *Aspergillus nidulans* TWY1.1 for producing fungi-derived secondary metabolite of polyketide neosartoricin B.

**Results:**

Polyketide-producing *A. nidulans* TWY1.1 substantially ameliorated the growth and the survival of sucrose-secreting cyanobacterium *S. elongatus* FL130 in salt-stressed environments. Besides sucrose, comparable amounts of other carbohydrates were released from axenically cultured FL130 cells, which could be efficiently consumed by TWY1.1. Relative to axenically cultured FL130, less glycogen was accumulated in FL130 cells co-cultured with TWY1.1, and the glycogen phosphorylase gene catalyzing the first step for glycogen degradation had two-fold expression. Different from axenically cultured filamentous fungi, abundant vacuoles were observed in fungal hyphae of TWY1.1 co-cultured with cyanobacterium FL130. Meanwhile, FL130 cells displayed a characteristic pattern of interacting with its heterotrophic partner, densely dispersing along certain hyphae of TWY1.1. Finally, polyketide neosartoricin B was produced from TWY1.1 in FL130-TWY1.1 co-cultures, which was tightly adjusted by nitrogen level.

**Conclusion:**

Overall, the results thoroughly proved the concept of pairing cyanobacteria with filamentous fungi to build artificial consortia for producing fungi-derived biomolecules.

**Supplementary Information:**

The online version contains supplementary material available at 10.1186/s13068-023-02283-6.

## Background

Environmental microbes typically live in complex communities, and each of them plays specialized roles, allowing niche differentiation and complementary functions that can enable more efficient resource utilization. One example of this is represented by lichens that are self-supporting symbioses composed of heterotrophs (mycobionts) and photoautotrophs (photobionts, cyanobacteria, and/or green algae) [1, 2]. Among them, photobionts supply organic carbon to the heterotrophic mycobionts, while the heterotrophs provide metabolites to support the health of the photosynthesizing partners [3–6]. Considering the numerous metabolites and the less reliance on exogenous nutrient sources relative to microbial mono-cultures, this natural consortium has considerable implications for the biotechnological opportunity to sustainably transform carbon dioxide and sunlight into bioproducts. Given the complexity in terms of the number of interacting species and the degree of connectivity between species in lichens, co-cultivation of an autotrophic species with a heterotrophic microbe is an alternative strategy that retains features of natural consortia, and increasing focus is being placed on organizing artificial microbial consortia for photobiological production of high-value compounds.

Screening of artificial cyanobacterial consortia at the laboratory scale is an invaluable requisite for the successful deployment of these consortia at the field scale. Some recent examples of artificial microbial consortia are based on cyanobacteria that have evolved oxygenic photosynthesis, an efficient system converting solar energy and CO_2_ into organic compounds. One of the critical requirements for generating cyanobacterial consortia is the capacity of a cyanobacterium to provide consumable organic carbon to its mycobiont partner. Considering the capability of naturally accumulating sucrose as a compatible solute [7], cyanobacteria have been engineered to export sucrose out by expressing the sucrose/proton symporter of *cscB* [8]. Extensive studies focus on the development of cyanobacteria as a novel source of sugar feedstock [9–11]. At the same time, many examples of artificial cyanobacterial consortia in the literature have shown that the heterotrophic partners can access the sole carbon source from the sucrose-secreting *Synechococcus elongatus* and transform the fixed carbon into more valuable bioproducts, including fatty acids [12], polyhydroxyalkanoate (PHA) [13], polyhydroxybutyrate (PHB) [14, 15], and secreted enzymes [16]. To be noticed, desired products in artificial cyanobacteria consortia are still limited, and enhancing the fitness of heterotrophic partners under co-culture conditions is still an urgent task to build an artificial consortium with better performance. Most of the heterotrophic partners involved in cyanobacterial consortia are unicellular yeast or bacteria cells, such as yeast *Rhodotorula glutinis* [12], and bacteria of *Pseudomonas putida* [13], *Halomonas boliviensis* [14], *Escherichia coli* [16], and *Bacillus subtilis* [16]. Until now, filamentous fungi are rarely evolved in artificial cyanobacterial consortia, which may contribute to several challenges in terms of morphology, pellet growth, and high viscosity. Nonetheless, filamentous fungi are well-known producers of a wealth of biochemicals, antibiotics, and other secondary metabolites with various biological activities. Many of these compounds, such as penicillin, cyclosporine, or lovastatin, are of great importance for human health. Moreover, filamentous fungi, such as *Aspergillus*, stand out from other microbial cell factories of bacterial or yeast origin due to their ability to consume various polymeric substrates [17] and tolerate extreme cultivation conditions [18, 19], especially in consideration of the moderate salt stress (100 to 200 mM NaCl) necessitating CscB-expressing cyanobacteria to steadily export sucrose. In fact, *Aspergillus* are closely related to lichen mycobionts, and the free-living *Aspergillus* species may have evolved from partners in lichens [20].

In this study, a cyanobacteria-fungi co-culture platform for bioproducts was constructed by a sucrose-secreting *S. elongatus* PCC 7942 (hereafter FL130) [21] and a filamentous fungus *Aspergillus nidulans*. As a proof of concept, polyketide neosartoricin B was produced from *A. nidulans* through heterologously expressing dermatophyte-derived polyketide synthase genes (hereafter TWY1.1) [22]. Polyketides are a structurally and functionally diverse group of natural products, some of which are potential sources for pharmaceuticals. Among them, neosartoricin B is a prenylated anthracenone that has the potential in inhibiting T-cell proliferation, suggesting its immunosuppressive application [22]. This study evaluated for the first time the capacity of artificial cyanobacterial consortia to produce fungi-derived secondary metabolites as represented by polyketides. Moreover, each partner’s behavior in physically integrated co-cultures was analyzed, in particular, the physiological adjustment of cyanobacterial cells would make itself more attractive as a photoautotrophic partner in artificial heterotroph–phototroph consortia, instead of organic carbon feedstock production in the photobiological platform.

## Results and discussion

### Growth of sucrose-secreting FL130 in mono-culture and co-culture paired with polyketide-producing TWY1.1

FL130 is an engineered *S. elongatus* 7942 that can secrete sucrose by IPTG-induced expression of sucrose transporter *cscB* and co-overexpression of native *sps* [21]. The *sps* gene encodes a natively fused protein of sucrose phosphate synthase and sucrose phosphate phosphatase, which are two inevitable enzymes in the main pathway for sucrose synthesis starting from fructose-6-phosphate and UDP-glucose [23]. In this study, FL130 cells were axenically cultured and co-cultured with filamentous fungus TWY1.1, respectively, in the BG-11[co] medium supplemented with NaCl at varied concentrations, with or without IPTG. Despite the formation of flocculants consisting of cyanobacterial cells and fungal mycelia, the majority of cyanobacterial cells were at suspended status in FL130-TWY1.1 co-cultures (data not shown). Thus, the expansion of non-flocculated FL130 was estimated by tracking the growth of suspended cyanobacterial cells after the removal of the flocculants.

**Fig. 1:**
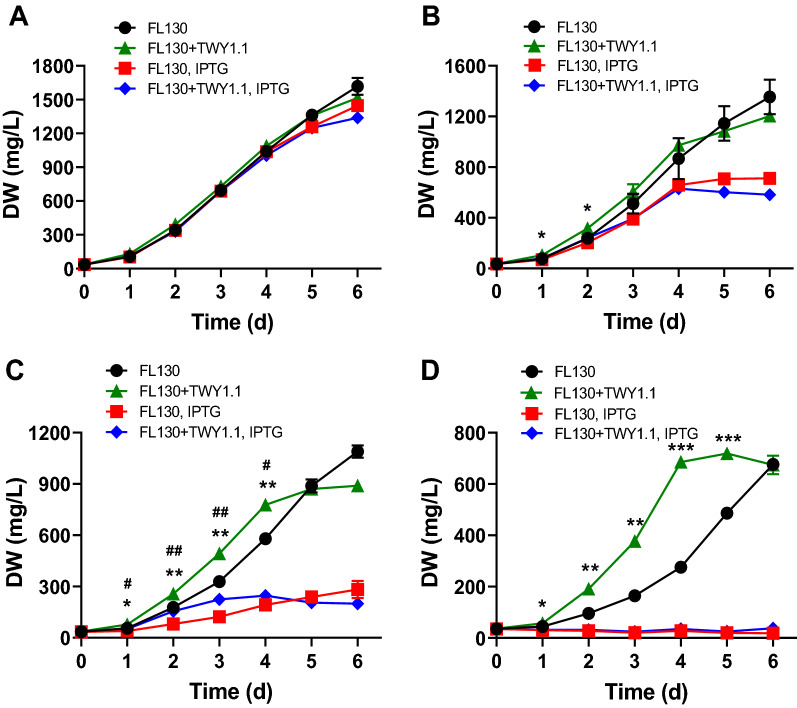
FL130 growth in mono-cultures and FL130-TWY1.1 co-cultures with or without IPTG supplementation. **A**–**D** FL130 growth in BG-11[co] medium added with 0 mM NaCl (**A**), 100 mM NaCl (**B**), 150 mM NaCl (**C**), and 200 mM NaCl (**D**). The *, **, and *** represent the significant difference of *p* < 0.05, *p* < 0.01, and *p* < 0.001, respectively, between axenic FL130 cultures and FL130-TWY1.1 co-cultures sampled at the same time without IPTG induction. # and ## represent the significant difference of *p* < 0.05 and *p* < 0.01, respectively, between axenic FL130 cultures and FL130-TWY1.1 co-cultures sampled at the same time with IPTG induction

With no salt added, FL130 grew well and exhibited a similar growth trend in mono-cultures and FL130-TWY1.1 co-cultures, no matter with or without IPTG induction (Fig. 1A). With salt addition, the growth of FL130 was significantly boosted in FL130-TWY1.1 co-cultures relative to mono-cultures when no IPTG was supplied (Fig. 1B–D). The specific growth rate of FL130 was 0.9/day and 0.78/day in the co-cultures, respectively, with NaCl concentrations of 150 and 200 mM, whereas it was 0.77/day and 0.54/day, respectively, in mono-cultures. Additionally, IPTG supply greatly reduced the accumulation of cyanobacterial biomass under salt stress, and the growth of FL130 in IPTG-added culture became much worse as NaCl concentration increased. The growth defeat obtained by IPTG addition under salt stress should be ascribed to the enhanced sucrose secretion, which contributes to a lower level of compatible solute in the cytoplasm to combat salt stress, as well as the divergent carbon flow from cytoplasm as part of cyanobacterial biomass to the extracellular environment. Particularly, FL130 cells failed to grow in mono- and co-cultures supplied with 200 mM NaCl and IPTG (Fig. 1D). In subsequent experiments for carbohydrate production and glycogen accumulation, this condition was not applied.

### Filamentous fungus TWY1.1 facilitated the growth and survival of cyanobacterium FL130 under fatal salt stress

**Fig. 2:**
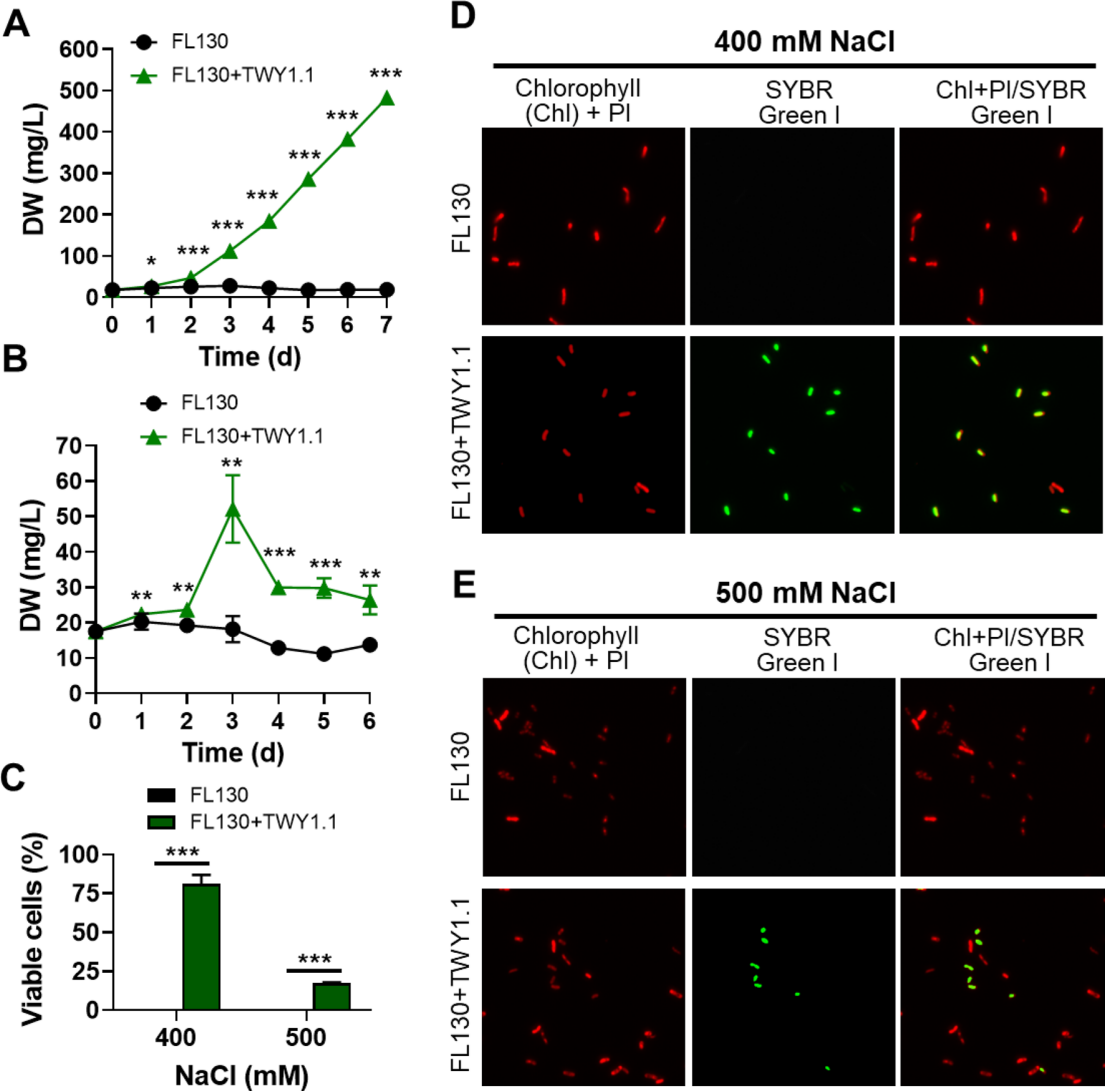
FL130 growth in mono-cultures and co-cultures in BG-11[co] supplied with NaCl in high concentration. **A**, **D** FL130 growth in BG-11[co] medium plus 400 mM NaCl; **B**, **E** FL130 growth in BG-11[co] medium plus 500 mM NaCl; **C** FL130 cell viability. Fluorescence microscopic images in **D** and **E** were for FL130 cells cultured for 6 days. The *, **, and *** represent significant difference of *p* < 0.05, *p* < 0.01, and *p* < 0.001, respectively

To further confirm the promoted growth of FL130 benefited from its heterotrophic partner under high salt stress conditions, the growth performance of FL130 cells was checked in mono- and co-cultures, after exposure to the even higher concentration of NaCl. As the results show, FL130 failed to grow in mono-cultures with salt stress of 400 and 500 mM NaCl (Fig. 2A, B). On the contrary, the co-cultured FL130 showed growth in these two salt conditions. Particularly, FL130 steadily grew with 400 mM NaCl, accumulating cell biomass close to 500 mg/L after 7 days. Viability assessment through the SYBR Green I/PI assay indicated viable FL130 cells presented in co-cultures with 400 and 500 mM NaCl (Fig. 2D, E), taking up 83 and 17% of total cyanobacterial cells, respectively (Fig. 2C). Conversely, no viable cyanobacterial cell was detected in the corresponding mono-cultures (Fig. 2D, E). The growth-promoting effect of filamentous *A. nidulans* was also observed on cyanobacterium *Nostoc* PCC 7413 in an unfavorable culture condition with low pH [24]. Future study is needed to explore the protective effect of *A. nidulans* for the growth of cyanobacteria under stressful conditions.

### Extracellular carbohydrates in mono-cultures of FL130 and co-cultures of FL130-TWY1.1 supplemented with NaCl at different concentrations

**Fig. 3:**
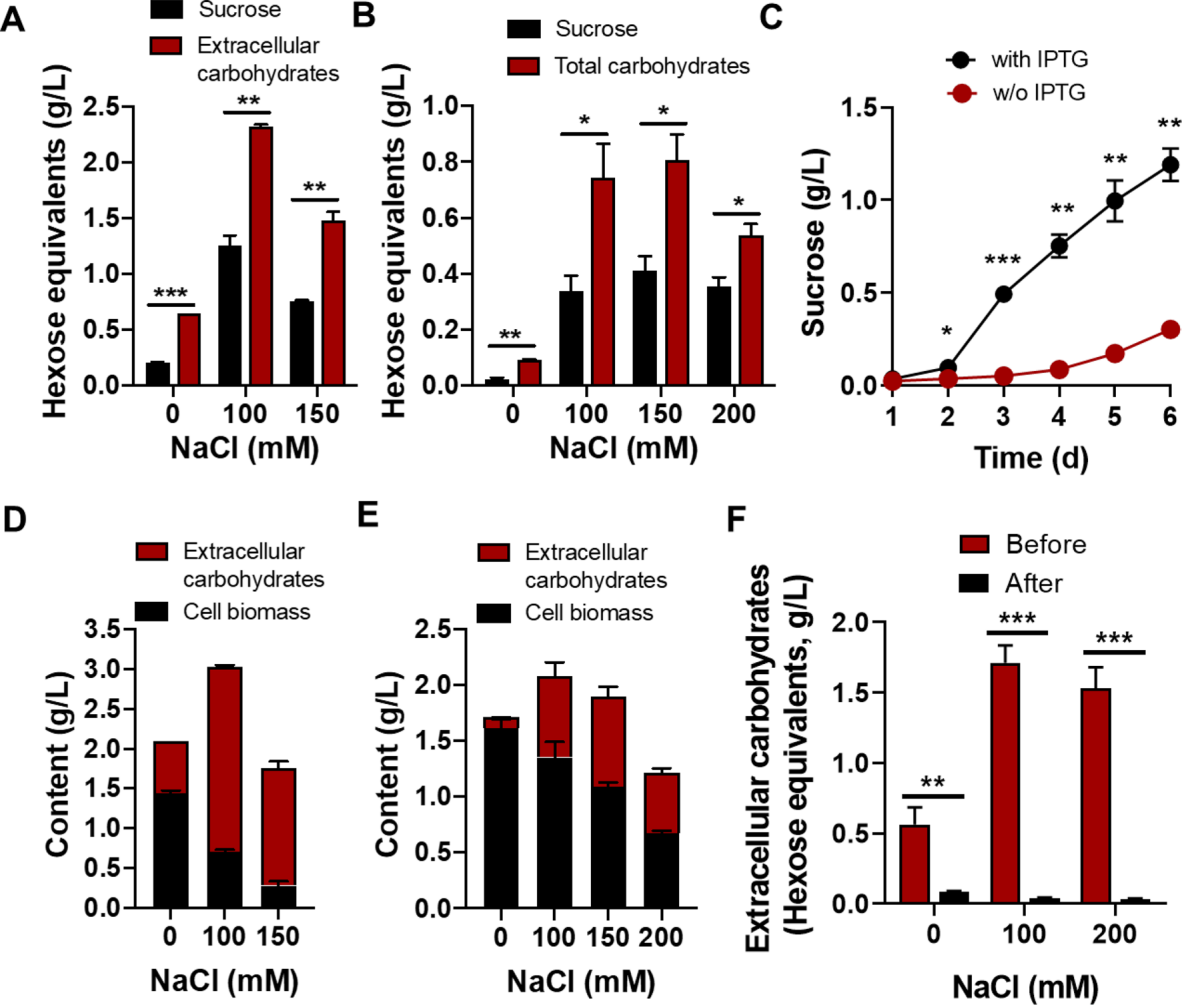
Carbohydrates production in axenic FL130 cultures. **A** Sucrose and total carbohydrate in axenic FL130 cultures added with IPTG; **B** Sucrose and total carbohydrate in axenic FL130 cultures without IPTG addition. **C** Sucrose in axenic FL130 cultures in BG-11[co] plus 100 mM NaCl, with or without IPTG. **D** Measurement of cell biomass and extracellular carbohydrates in axenic FL130 cultures added with IPTG. **E** Measurement of cell biomass and extracellular carbohydrates in axenic FL130 cultures without IPTG. **F** Extracellular carbohydrates in the spent medium of axenic FL130 culture before and after inoculation with TWY1.1 spores. The spent medium of axenic FL130 cultures with variable salt (0, 100, or 200 mM NaCl) were collected at 6 days and inoculated with TWY1.1 spores. Subsequently, the contents of total carbohydrates were measured after 2 days of cultivation. The *, **, and *** represent significant difference of *p* < 0.05, *p* < 0.01, and *p* < 0.001, respectively

FL130 was engineered to secrete sucrose by IPTG-induced expression of the native *sps* and *E.coli*-derived *cscB*. Here, sucrose production was monitored in FL130 mono-cultures. Generally, the contents of extracellular sucrose were significantly elevated when FL130 cells were exposed to salt stress, no matter with or without IPTG induction (Fig. 3A, B). Levels of extracellular sucrose under each NaCl condition were much higher with IPTG induction, particularly in 100 mM NaCl condition. Sucrose was steadily accumulated in 100 mM NaCl-supplemented FL130 mono-cultures, reaching the level of 1.2 g/L with IPTG induction and 0.3 g/L without IPTG induction after 6 days (Fig. 3C).

Considering sucrose should not be the only carbohydrate secreted by FL130, contents of total extracellular carbohydrates were measured concurrently in axenic FL130 cultures exposed to various salt stress. Compared with the levels of extracellular sucrose, hexose equivalents of extracellular carbohydrates were 3.3-, 2.0-, and 2.1-fold in 0, 100, and 150 mM NaCl conditions in the presence of IPTG, respectively (Fig. 3A); meanwhile, they were 4.5-, 2.4-, 2.1-, and 1.6-fold in 0, 100, 150, and 200 mM NaCl conditions in the absence of IPTG, respectively (Fig. 3B). The above results indicate the presence of a large proportion of extracellular non-sucrose carbohydrates in axenic FL130 culture. Generally, most cyanobacteria produce extracellular polymeric substances mainly constituted by heteropolysaccharides. Heteropolysaccharides of cyanobacteria are documented with composition mainly consisting of xylose, arabinose, galactose, rhamnose, sucrose, maltose, glucose, and fructose [25].

Since exported carbohydrates principally represent fixed carbon that is diverted from the cell growth process, we then monitored carbon distribution between cyanobacterial biomass and extracellular carbohydrates. In the presence of IPTG, a major fraction of organic carbon was secreted extracellularly under salt stress, accounting for 327 and 528% of cell biomass, respectively, in 100 and 150 mM NaCl conditions (Fig. 3D). In the absence of IPTG, extracellularly accumulated carbohydrates in axenic FL130 culture took up 54, 74, and 80% of cell biomass in 100, 150, and 200 mM NaCl conditions, respectively (Fig. 3E).

The release of a fraction of photosynthetically fixed organic matter into the environment during growth is one characteristic of photosynthesizing microorganisms, and dissolved organic carbon (DOC) values are reported to range from 5 to 50% of that fixed by photosynthesis [26]. *Synechococcus elongatus*, capable of naturally accumulating intracellular sucrose as a compatible solute, is extensively manipulated to secrete sucrose by heterologously expressing *cscB* [8–10, 27], which is deemed to be potentially an attractive alternative to plant crops for sucrose generation. However, few studies get a glimpse of extracellular carbohydrates other than sucrose. Here, we found that the levels of total extracellular carbohydrates were approximately two times sucrose levels in the spent medium of axenic FL130 cultures supplemented with NaCl (Fig. 3A, B), which indicates half of the fixed carbon was lost if only sucrose were harvested. Contrary to recovering sucrose from the cultural system, pairing a heterotrophic partner represents an alternative strategy to directly utilize sucrose in the culture.

### Filamentous fungus TWY1.1 efficiently consumed FL130-derived extracellular carbohydrates and accumulated fungal biomass in FL130-TWY1.1 co-cultures

To evaluate the capacity of filamentous fungus TWY1.1 for consuming sucrose and other carbohydrates from cyanobacterium FL130, we inoculated TWY1.1 spores into the spent medium of axenic FL130 cultures with different NaCl concentrations. The growth of TWY1.1 led to efficient consumption of organic carbohydrates, draining approximately 98% extracellular carbohydrates in NaCl-supplied FL130 spent medium (Fig. 3F). Filamentous fungus TWY1.1 also demonstrated its superb organic carbon-consuming capacity in FL130-TWY1.1 co-cultures, leaving a minute amount of extracellular carbohydrates detected (Additional file 1: Fig. S1). It was further proved by the remarkably low level of total organic carbon in the supernatants of FL130-TWY1.1 co-cultures. The superior capacity of *A. nidulans* for consuming cyanobacteria-derived extracellular carbohydrates did not present in the culture of *A. nidulans* paired with *Nostoc* PCC 7413, within which a large fraction of extracellular polysaccharides released from cyanobacteria could not be assimilated by the heterotrophic partner *A. nidulans* [24]. A variety of genes encoding carbohydrate active enzymes and the complementary function assignment indicate the potential for polysaccharide-degrading capacity of *A. nidulans*. However, enzymes with the same general activity are identified to exhibit significant differences in substrate specificity [28], which would result in differences in polysaccharide degradation and utilization.

### Glycogen accumulation of FL130 in mono- and co-cultures

**Fig. 4:**
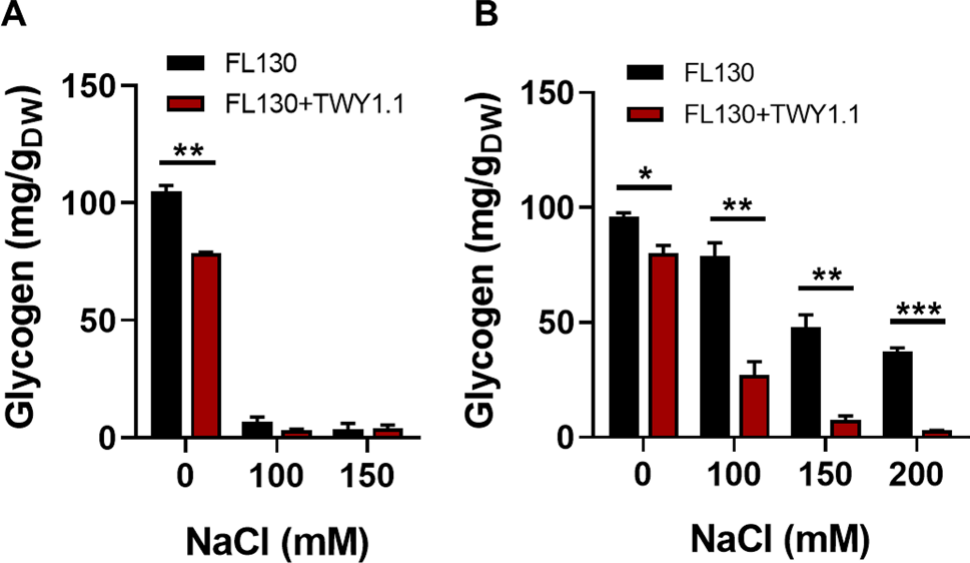
Glycogen accumulation of FL130 in mono-cultures and FL130-TWY1.1 co-cultures. **A** Cultures added with IPTG; **B** Cultures without IPTG. The *, **, and *** represent significant difference of *p* < 0.05, *p* < 0.01, and *p* < 0.001, respectively

Cyanobacteria naturally synthesize and accumulate large amounts of compatible solutes, such as sucrose, when exposed to salt stress [7]. Meanwhile, cyanobacterial cells also store abundant carbon and energy from photosynthesis in the form of glycogen, a high molecular branched α-polyglucan [29]. In NaCl-free conditions, axenically cultured FL130 accumulated the highest amount of glycogen, with 105 mg/g dry weight (g_DW_) in the presence of IPTG and 96 mg/g_DW_ in the absence of IPTG (Fig. 4). In NaCl-supplemented conditions, glycogen contents of IPTG-induced FL130 decreased to less than 5 mg/g_DW_ in mono- and co-cultures (Fig. 4A). However, with no IPTG addition, glycogen contents gradually decreased in axenic FL130 cells as salt stress increased, with 37 mg/g_DW_ remaining in 200 mM NaCl condition (Fig. 4B). Moreover, glycogen content significantly declined in co-cultured FL130 cells in various NaCl conditions compared with axenically cultured cells. To be specific, the levels of glycogen in co-cultured FL130 cells were 34, 16, and 8% of those in the corresponding axenic FL130 cultures supplemented with 100, 150, and 200 mM NaCl. Sucrose consumption by TWY1.1 in the co-culture likely accelerated sucrose discharge from cyanobacteria due to its concentration difference between the intracellular and extracellular environment, which then induced glycogen degradation to provide sucrose for stress tolerance.

Glycogen synthesis is closely linked to photosynthesis carbon output, with photosynthetically fixed carbon in excess diverted to glycogen storage as the carbon and electron sources for cellular respiration in the dark. Besides, glycogen metabolism is also an important cellular energy buffer, contributing to energy balancing in cyanobacteria [30]. It has been reported that salt stress significantly inhibits the photosynthetic activity of cyanobacteria [31]. The decreased glycogen accumulation accompanied by increased salt stress is probably a direct result of declined photosynthetic activity, which would provide less excess carbon and energy to store in glycogen.

**Fig. 5:**
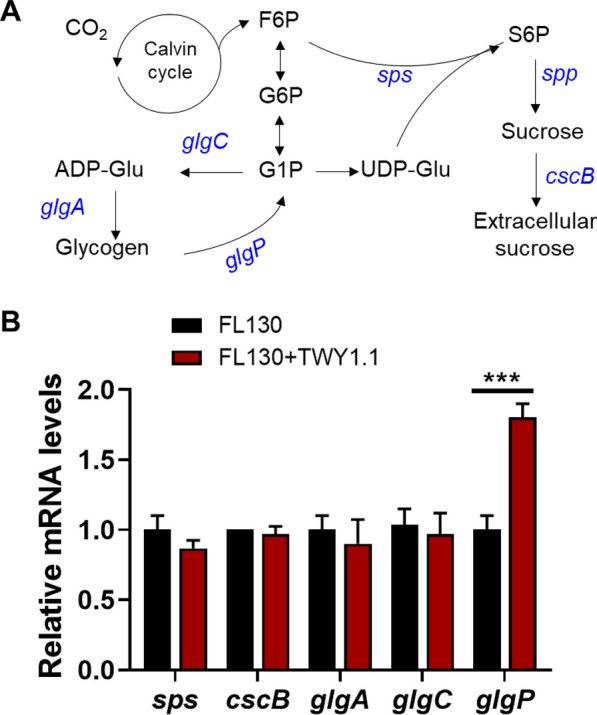
Sucrose and glycogen synthesis in *S. elongatus* FL130. **A** Schematic representation of sucrose and glycogen synthesis in *S. elongatus* FL130. The sucrose permease CscB was expressed for sucrose excretion. Genes highlighted in blue encode enzymes for glycogen and sucrose production. In the genome of *S. elongatus*, the *sps* encodes a combined SPS/SPP fusion protein. **B** RT-PCR for monitoring gene expression in sucrose and glycogen synthesis for FL130 in axenic culture and FL130-TWY1.1 co-culture in BG-11[co] medium added with 100 mM NaCl. The *** represents significant difference of *p* < 0.001

Glycogen accumulation in cyanobacterial cells is the result of two opposite processes, including glycogen synthesis and glycogen consumption. As shown in Fig. 5A, ADP-glucose pyrophosphorylase (*glgC*) catalyzes the reaction from glucose 1-phosphate (G1P) to ADP-glucose, and glycogen synthase (*glgA*) converts ADP-glucose into glycogen. Glycogen phosphorylase (GlgP) initiates the first step of glycogen degradation, generating G1P, which is then converted to UDP-glucose, one of the precursors for sucrose synthesis. To figure out the causation of declined glycogen content in FL130 co-cultured with a fungal partner, the expression of genes involved in these processes was quantified. Compared with mono-cultures, genes of *glgC* and *glgA* involved in glycogen synthesis displayed similar expression levels in FL130 co-cultured with TWY1.1, whereas the *glgP* gene, expressing an enzyme catalyzing the first step for glycogen degradation, showed approximately two-fold of expression in 100 mM NaCl co-culture condition without IPTG (Fig. 5B). The overexpressed *glgP* is recently demonstrated to facilitate glycogen degradation and result in increased sucrose production in *S. elongatus* PCC 7942 [32]. As a heterotrophic partner, filamentous fungus TWY1.1 seems to be a carbon sink for cyanobacterium FL130, making the phototrophic partner convert more polymer carbohydrates (glycogen) into transportable sugars, such as sucrose.

### Phenotypic characteristics and biomass accumulation of FL130 and TWY1.1 in FL130-TWY1.1 co-cultures

**Fig. 6:**
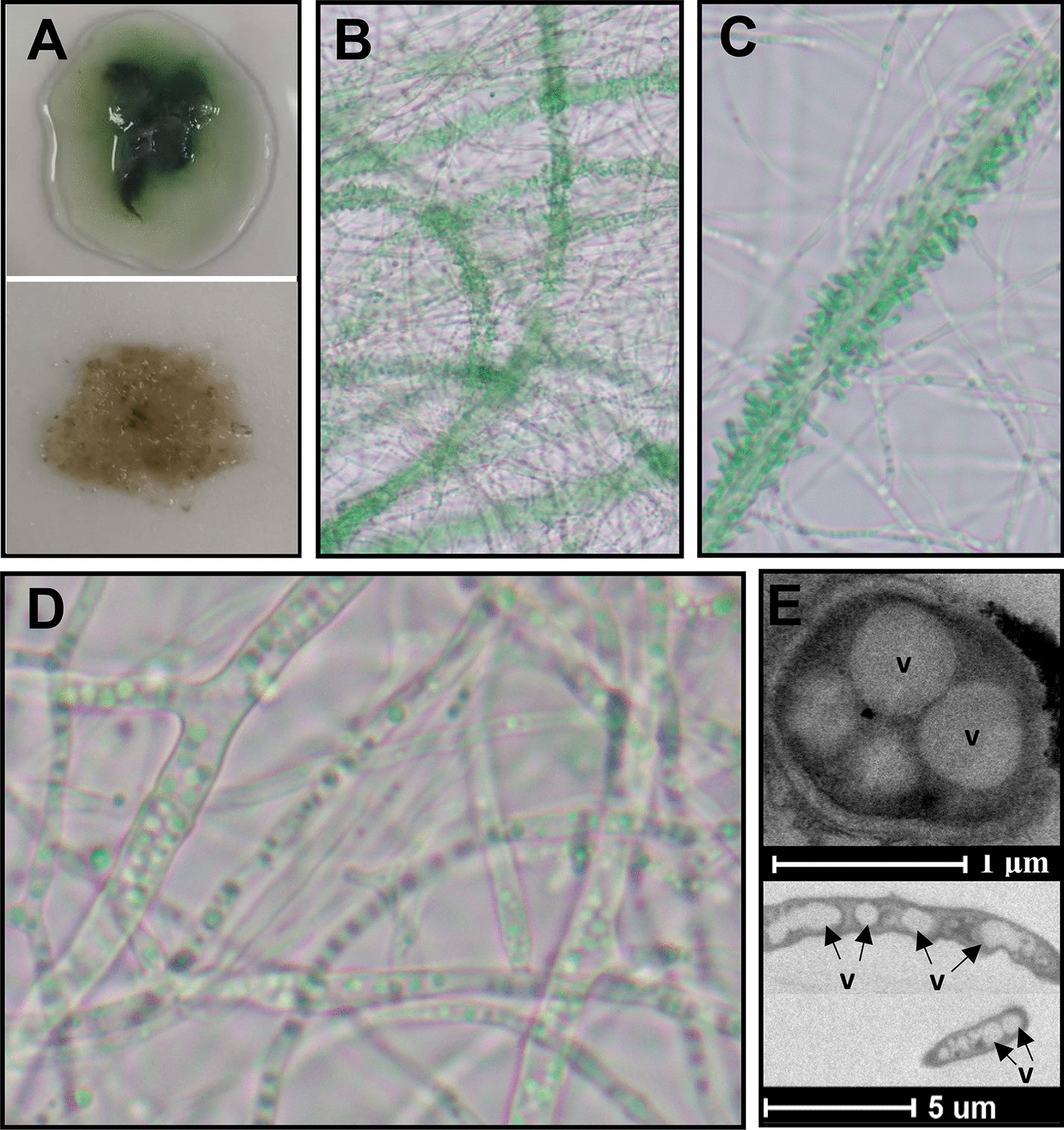
Images for FL130-TWY1.1 co-cultures in 100 mM NaCl-added BG-11[co] medium. **A** FL130-TWY1.1 co-culture pellets; **B**–**D** FL130-TWY1.1 co-culture pellets observed under a light microscope, with 200× magnification for (**B**), 400× magnification for **C**, and 1000× magnification for **D**; **E** TEM of fungal hyphae from co-culture pellets after flushing with distilled water. The letter “v” indicated vacuoles

In FL130-TWY1.1 co-cultures, filamentous fungus TWY1.1 could grow into mycelia by exploiting external carbon sources from the co-cultured photoautotrophic partner FL130. The fungal mycelia, together with cyanobacterial cells, formed flocculants in the co-culture, which displayed as green clumps (Fig. 6A, top one). Interestingly, cyanobacterium FL130 cells in the green clumps densely attached to certain fungal hyphae instead of randomly dispersed in the whole pellets (Fig. 6B, C). Fungal hyphae in co-cultured flocculants were extensively vacuolated, with large amounts of vacuoles filled in hyphal space (Fig. 6D). Vacuoles could also be visualized by transmission electron microscopy (Fig. 6E). In contrast, vacuoles could be barely observed in the hyphae of TWY1.1 cultured axenically (Additional file 2: Fig. S2). As a vital organelle at the heart of fungal physiology, vacuoles may play an important role in fungal growth and differentiation in co-culture environments.

The accumulation of fungal biomass in FL130-TWY1.1 co-cultures was confirmed based on the above results. To quantify fungal biomass, the green clumps of FL130-TWY1.1 flocculants were flushed with distilled water to remove attached cyanobacterial cells until it changed into brown pellets, with only fungal mycelia left (Fig. 6A, bottom one). Considerable amounts of fungal biomass were accumulated in the co-cultures, especially supplemented with 150 mM NaCl and 100 mM NaCl plus IPTG, which accounted for 31 and 40% of cyanobacterial cell biomass in the corresponding cultures, respectively (Table 1). The substantial expansion of filamentous fungus TWY1.1 proved the efficient secretion of organic carbon from FL130 in FL130-TWY1.1 co-cultures. Furthermore, the expression level of *sps* and *cscB* in axenic and co-cultured FL130 displayed no significant difference in the same cultural condition (Fig. 5B), which indicated the comparable sucrose-secreting capacity in these two culture conditions.

**Table 1:** Biomass dry weight (DW) of suspended FL130 and TWY1.1 pellets in the co-cultures

NaCl (mM)	DW of suspended FL130 (mg/L)		DW of TWY1.1 pellets (mg/L)	
	+ IPTG	− IPTG	+ IPTG	− IPTG
0	1339.5 ± 35.8	1515.2 ± 87.3	93.8 ± 8.8	26.6 ± 2.2
100	582.7 ± 26.9	1203.3 ± 26.9	231.3 ± 17.7	181.3 ± 8.8
150	199.5 ± 9.9	889.8 ± 9.0	78.1 ± 0.0	275 ± 0.0
200	ND	674.5 ± 35.8	ND	150 ± 8.8

*ND* not detected

In this study, even a trace amount of sucrose from non-induced FL130 cells due to leaky expression of *cscB*, together with minute other extracellular carbohydrates, could support fungal spore germination and mycelia formation in the co-culture. However, certain heterotrophic partners in co-cultures with sucrose-secreting *S. elongatus* failed to proliferate without IPTG addition [13, 16]. Those heterotrophic microbes displayed limited sucrose-consuming capability. For example, round yeast *Saccharomyces cerevisiae* could grow only when sucrose concentrations were higher than 2.5 g/L [16]. Any non-recovered carbon from the photoautotrophic partner would thus constitute a loss factor in light energy conversion and represent an unexplored resource. Considering the high cost of IPTG and its detrimental effect on cell growth as displayed in Fig. 1, heterotrophic partner’s growth and synthesis of desired products without IPTG addition was a more desirable character for application as synthetic consortia in practical use.

### Production of neosartoricin B in FL130-TWY1.1 co-cultures

**Fig. 7:**
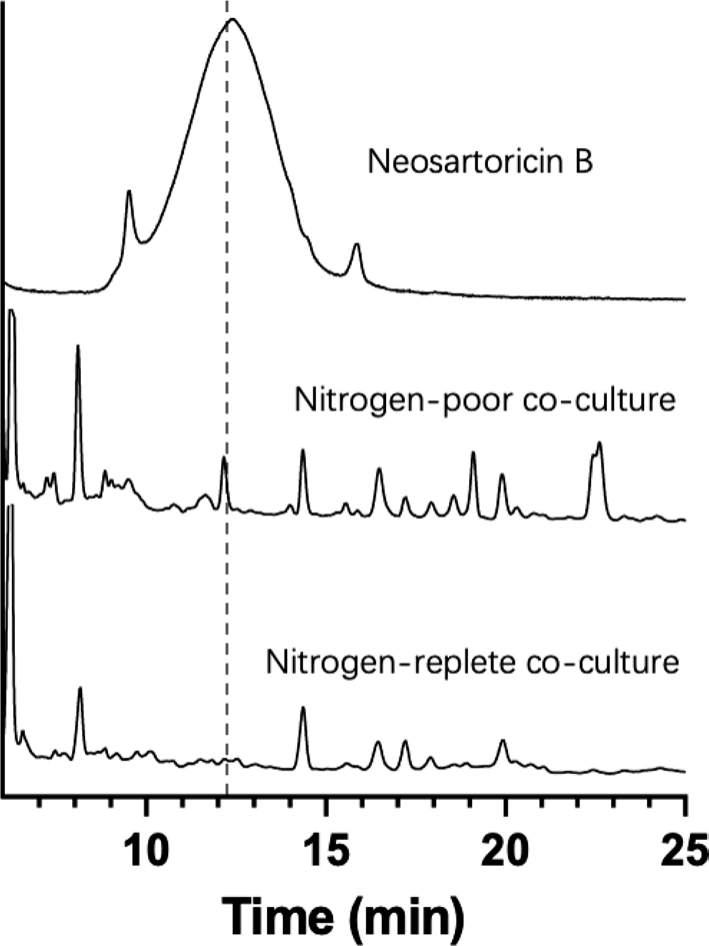
Production of neosartoricin B from FL130-TWY1.1 co-cultures. HPLC detection of neosartoricin B (400 nm). Trace 1: neosartoricin B standard; Trace 2: Organic extracts from nitrogen-poor FL130-TWY1.1 co-cultures; Trace 3: Organic extracts from nitrogen-replete FL130-TWY1.1 co-cultures

Nitrogen is one external signal frequently observed in regulating the biosynthesis of secondary metabolites in fungi [33]. Here we included two nitrogen levels in FL130-TWY1.1 co-cultures in BG-11[co] medium supplemented with 150 mM NaCl but without IPTG: nitrogen-poor condition (0.3 g/L NaNO_3_) and nitrogen-replete condition (3 g/L NaNO_3_). The majority of nitrogen remained in nitrogen-replete co-cultures, whereas nitrogen was consumed completely after 4 days in nitrogen-poor co-cultures (Additional file 3: Fig. S3). HPLC analyses showed that neosartoricin B was produced in nitrogen-poor co-cultures, with the level of 0.2 mg/L detected. In contrast, neosartoricin B was barely produced in nitrogen-replete co-cultures (Fig. 7), although fungal biomass accumulation was similar in both nitrogen-replete and nitrogen-poor conditions (Additional file 3: Fig. S3). Thus, nitrogen level was a critical factor in producing heterotrophically expressed secondary metabolite neosartoricin B in *A. nidulans* co-cultured with cyanobacterial cells.

Compared to traditional fermentation with a single microorganism, the use of filamentous fungi paired with cyanobacteria as a production platform for secondary metabolites is still in its infancy, and with the growing interest in using artificial heterotroph–phototroph consortia for producing a wide variety of compounds, more work is expected to optimize the cultivation system.

## Conclusions

The present study demonstrated the feasibility of pairing sucrose-secreting cyanobacterial cells with filamentous fungi to build artificial consortia for producing fungi-derived polyketide. During this process, the boosted growth and the survival of cyanobacterial cells afforded by the filamentous heterotrophic partner, the efficient consumption of cyanobacteria-released carbohydrates by filamentous fungus, fewer polymer carbohydrates stored as glycogen in cyanobacterial cells, and polyketide neosartoricin B production by filamentous fungi all pointed to the promise of cyanobacteria-fungi co-culture system as a novel production platform for secondary metabolites.

## Material and methods

### Strains and growth conditions

The sucrose-secreting *Synechococcus elongatus* FL130 (FL130) was a generous gift from Prof. Xuefeng Lu (Qingdao Institute of Bioenergy and Bioprocess Technology, Chinese Academy of Sciences, China). FL130 was an engineered cyanobacterium capable of co-overexpressing *cscB* (encoding a sucrose transporter) and native *sps* (encoding a natively fused protein of sucrose phosphate synthase SPS and sucrose phosphate phosphatase SPP), with chloramphenicol resistance and spectinomycin resistance. The expression of both *cscB* and *sps* was driven by isopropyl-d-1-thiogalactopyranoside (IPTG)-inducible promoters [21]. For standard cultivation, FL130 cells were grown in 100 mL Erlenmeyer flasks containing 40 mL BG-11 medium supplemented with 10 μg/mL chloramphenicol and 20 μg/mL spectinomycin. Flasks were incubated in a horizontal rotary shaker at 100 rpm and 30 °C, supplied with 1% CO_2_ (v/v). Illumination was provided by cool-white fluorescent lamps to give a light intensity of 100 μmol m^−2^ s^−1^ with a 16:8 h light/dark cycle.

The polyketide neosartoricin B-producing fungal strain *Aspergillus nidulans* TWY1.1 (TWY1.1) [22] was grown at 37 °C on glucose minimum medium (GMM) [34] supplemented with 0.5 μM pyridoxine HCl. Spores were collected from GMM agar plates in sterile water, filtered, and maintained as a suspension at 4 °C for up to one month.

Co-culture medium (BG-11[co]) of FL130 and TWY1.1 was designed based on BG-11. Compared to BG-11, BG-11[co] contains 3 g/L NaNO_3_, fivefold of KH_2_PO_4_, fivefold of MgSO_4_, fivefold of trace elements, 0.5 μM pyridoxine HCl, and 3 g/L HEPES (pH 8.0). For mono-cultures of FL130 and co-cultures of FL130 together with TWY1.1, exponential-phase FL130 cells (1.6 × 10^7^ per mL in initial culture) and TWY1.1 spore suspension (1 × 10^5^ spores per mL in initial culture) were transferred into each flask containing BG-11[co] medium, which was supplemented with NaCl in various concentrations. If necessary, 1 mM IPTG was also supplied. Flasks with cultures were weighed before incubation and were added with distilled water before each sampling to correct for water evaporation. Cultures were incubated at 35 °C, 100 rpm, and 1% CO_2_, under the illumination of 100 μmol photons m^−2^ s^−1^ with a 16:8 h light/dark cycle.

### Quantification of *S. elongatus* FL130 and *A. nidulans* TWY1.1

Cell growth of FL130 was monitored by measuring the optical density at a wavelength of 750 nm (OD_750_) and converted to biomass dry weight (DW) with a pre-established calibration between the OD_750_ and DW of FL130 cultures (1.0 OD_750_ unit equals approximately 633.32 mg L^−1^ DW). For FL130 suspension cells in FL130-TWY1.1 co-cultures, cultures were filtered through Miracloth (Millipore, MA, USA), and OD_750_ was then measured. For cell biomass in the final cultures, FL130 mono-cultures were harvested by centrifugation and washed three times with distilled water. FL130 suspension cells in FL130-TWY1.1 co-cultures were filtered through Miracloth first, then washed. The *A. nidulans* TWY1.1 pellets (the solid content intercepted on the Miracloth) were washed with distilled water. Water-washed biomass was all lyophilized by a vacuum freeze drier and weighed.

### Microscopy techniques

The SYBR Green I/PI assay was performed to determine the live and dead cells of FL130, as previously described [35]. Briefly, SYBR Green I (10,000× stock, Invitrogen) was mixed with propidium iodide (PI, 20 mM, Sigma) into sterile distilled H_2_O and vortexed thoroughly. The staining mixture was added to 50 μL FL130 cultures in 1.5 mL Eppendorf tubes, mixed thoroughly, and incubated at room temperature in the dark for 15 min. Specimens of FL130 cells were examined on an Olympus IX71 inverted fluorescence microscope system, and images were captured. Image analysis was performed using ImageJ, with green-colored cells representing live cells and red-colored cells representing total cells. The viability of FL130 cells was calculated by dividing the live cell count by the total cell count. Interaction and symbiosis between FL130 and TWY1.1 were examined with a light microscope. Images were taken from the cyanobacteria-fungi aggregates after 6 days of co-cultivation.

### Content determination of extracellular sucrose and extracellular carbohydrates

For sucrose determination, 1 mL of FL130 culture or FL130-TWY1.1 co-culture grown in the flasks under different conditions (see above) was sampled at designated time points and then centrifuged at 10,000*g* for 10 min. The amount of sucrose in the supernatant was analyzed using a sucrose/d-glucose assay kit (Megazyme, Ireland) according to the manufacturer’s instructions. Contents of total extracellular carbohydrates in the supernatant were quantified by colorimetric assay on 96-well plate at 620 nm wavelength. The process of the color reaction was carried out according to Yemm and Willis [36], and pure glucose was used as the standard.

### Determination of glycogen content in *S. elongatus* FL130 cells

Glycogen was isolated as previously reported with minor modifications [37]. Briefly, FL130 cultures were centrifugated at 12,000*g* for 10 min. The harvested cell pellets were washed three times with sterile water and then suspended in 30% (w/v) KOH. After incubation at 95 °C for 2 h, ice-cold ethanol was added to a final concentration of 75% (v/v) for glycogen precipitation, followed by incubation on ice for at least 2 h. The glycogen pellets were obtained after a centrifugation step (10 min, 4 °C, 10,000*g*), washed with 70 and 98% (v/v) ethanol one after another and dried in a SpeedVac.

The isolated glycogen pellets were re-suspended in 100 mM sodium acetate (pH 4.5) and enzymatically hydrolyzed to glucose by treatment with amyloglucosidase (Megazyme, Ireland) at 60 °C for 2 h. Glycogen content was determined by monitoring glucose concentration with a sucrose/D-glucose assay kit (Megazyme, Ireland) according to the manufacturer’s instructions.

### Transmission electron microscopy

Fungal pellets were fixed overnight at 4 °C in 0.01 M PBS (pH 7.4) supplemented with 2.5% glutaraldehyde and then washed three times with 0.01 M PBS (pH 7.4), followed by post-fixation in 1% osmium tetroxide. Graded ethanol was used to dehydrate the cells, after which they were embedded in epoxy resin. Resin blocks were cut into ultrathin sections and stained with uranyl acetate and lead citrate. The ultrastructure of fungal hypha was examined by transmission electron microscopy (TEM) using a JEOL-1230 transmission electron microscope (JEOL, Tokyo, Japan).

### Transcript analysis by RT-PCR

Total RNA was extracted by using the TRI Reagent (Molecular Research Center). Single-strand cDNA was synthesized with Hifair III 1st-strand cDNA Synthesis SuperMix for qPCR (gDNA digester plus) (Yeasen, China) according to the manufacturer’s instruction. RT-PCR was performed using Hieff UNICON Universal Blue qPCR SYBR Green Master Mix (Yeasen, China) to analyze the expression of *ppc* (as a reference gene), *sps*, *cscB*, *glgA*, *glgC*, and *glgP*, with the QuantStudio 3 Real-Time PCR System (Applied Biosystems, USA). The RT-qPCR primer sequences used in this study are shown in Additional file 4: Table S1. The relative abundance of different mRNA molecules was estimated using 2^−ΔΔCT^.

### Neosartoricin B content analysis

Neosartoricin B in the culture was extracted as described previously [22] with minor modification. Briefly, FL130-TWY1.1 co-cultures were taken, filtered through Miracloth, and centrifugated at 12,000*g* for 10 min. Neosartoricin B was extracted from supernatant with ethyl acetate (EtOAc)/methanol (MeOH)/acetic acid (AcOH) (89:10:1). The organic phase was evaporated to dryness and redissolved in MeOH. Then 20 μL dissolved extract was injected for HPLC analysis. Neosartoricin B quantification in samples was carried out with an Agilent 1220 Infinity II HPLC according to the standard curve established with the neosartoricin B standard (BioAustralis, Australia) (Additional file 5: Fig. S4).

### Statistical analysis

Three biological replicates were performed for all data collection, and the statistical tests for significance were determined via a two-tailed *t* test using Excel software.

## Supplementary Information

**Additional file 1: Figure S1.** Total carbohydrates in the supernatants of FL130-TWY1.1 co-cultures.

**Additional file 2: Figure S2.** Images for axenic cultured TWY1.1 in BG-11[co] medium added with 100 mM NaCl and 2.5 g L^-1^ glucose.

**Additional file 3: Figure S3.** The FL130-TWY1.1 co-cultures in nitrogen-poor and nitrogen-replete BG-11[co] medium.

**Additional file 4: Table S1.** RT-PCR primer sets used in this study.

**Additional file 5: Figure S4.** Standard curve of neosartoricin B.

## Data Availability

All data generated or analyzed during this study are included in this published article (and its Additional files).

## Abbreviations

IPTG: Isopropyl-d-1-thiogalactopyranoside
*ppc*: Phosphoenolpyruvate carboxylase
ADP-Glu: Adenosine diphosphate glucose
F6P: Fructose 6-phosphate
G1P: Glucose 1-phosphate
G6P: Glucose 6-phosphate
S6P: Sucrose 6-phosphate
UDP-Glu: Uridine diphosphate glucose
*cscB*: Sucrose permease
*glgA*: Glycogen synthase
*glgC*: ADP-glucose pyrophosphorylase
*spp*: Sucrose phosphate phosphatase
*sps*: Sucrose phosphate synthase
*glgP*: Glycogen phosphorylase
