# Supplementary material for: Generation and comprehensive analysis of Synechococcus elongatus–Aspergillus nidulans co-culture system for polyketide production
Source: Biotechnol Biofuels Bioprod. 2023 Mar 1;16:32. doi: 10.1186/s13068-023-02283-6 (PMC9979520; doi:10.1186/s13068-023-02283-6)
Supplement: Supplementary file 1 — Additional file 1: Figure S1. Total carbohydrates in the supernatants of FL130-TWY1.1 co-cultures. [file 13068_2023_2283_MOESM1_ESM.docx]

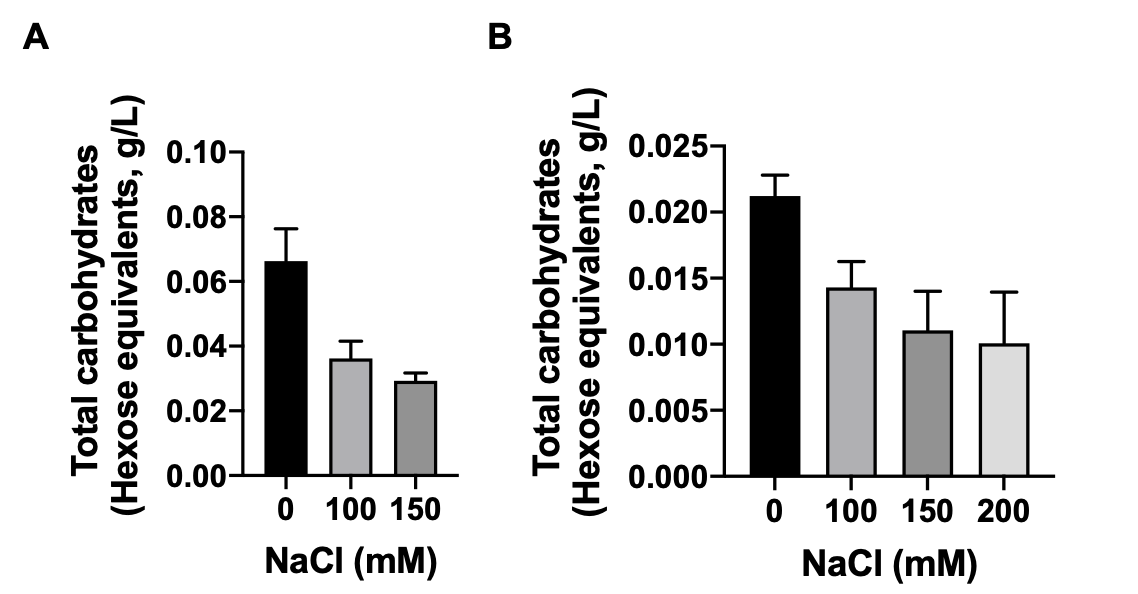


**Figure S1. Total carbohydrates in the supernatants of FL130-TWY1.1 co-cultures.** (A) Cultures added with IPTG; (B) Cultures without IPTG.
