# Supplementary material for: Generation and comprehensive analysis of Synechococcus elongatus–Aspergillus nidulans co-culture system for polyketide production
Source: Biotechnol Biofuels Bioprod. 2023 Mar 1;16:32. doi: 10.1186/s13068-023-02283-6 (PMC9979520; doi:10.1186/s13068-023-02283-6)
Supplement: Supplementary file 2 — Additional file 2: Figure S2. Images for axenic cultured TWY1.1 in BG-11[co] medium added with 100 mM NaCl and 2.5 g L-1 glucose. [file 13068_2023_2283_MOESM2_ESM.docx]

**A**

**B**


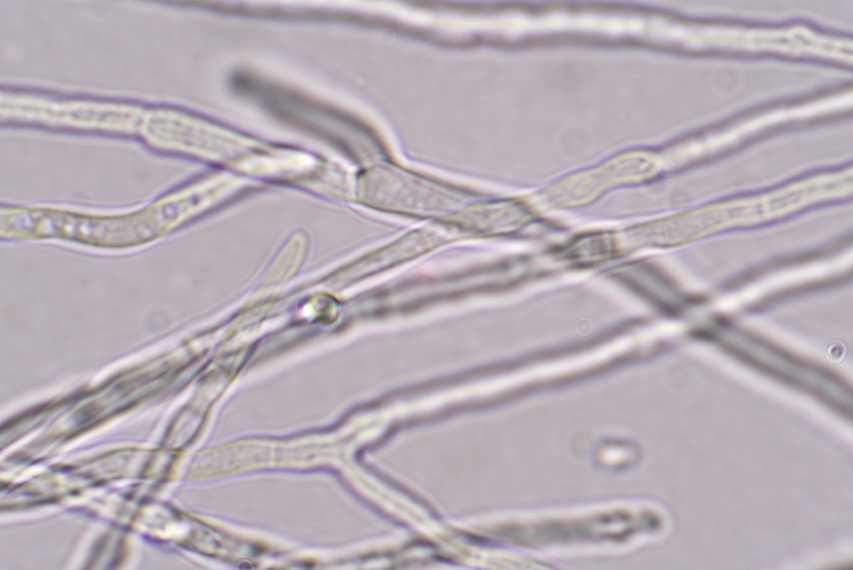


**Figure S2.** Images for axenic cultured TWY1.1 in BG-11[co] medium added with 100 mM NaCl and 2.5 g L^-1^ glucose. (A) 400x magnification; (B) 1000x magnification.
