# Supplementary material for: Generation and comprehensive analysis of Synechococcus elongatus–Aspergillus nidulans co-culture system for polyketide production
Source: Biotechnol Biofuels Bioprod. 2023 Mar 1;16:32. doi: 10.1186/s13068-023-02283-6 (PMC9979520; doi:10.1186/s13068-023-02283-6)
Supplement: Supplementary file 3 — Additional file 3: Figure S3. The FL130-TWY1.1 co-cultures in nitrogen-poor and nitrogen-replete BG-11[co] medium. [file 13068_2023_2283_MOESM3_ESM.docx]

**Figure S3.** **The FL130-TWY1.1 co-cultures in nitrogen-poor (0.3 g/L NaNO_3_) and nitrogen-replete (3 g/L NaNO_3_)** BG-11[co] medium. (A) Nitrogen consumption in FL130-TWY1.1 co-cultures as time course; (B) Biomass dry weight (DW) of suspended FL130 and TWY1.1 in FL130-TWY1.1 co-cultures. The ** represents significant difference of p < 0.01. The “NS” indicates no significant difference.
