# Supplementary material for: Generation and comprehensive analysis of Synechococcus elongatus–Aspergillus nidulans co-culture system for polyketide production
Source: Biotechnol Biofuels Bioprod. 2023 Mar 1;16:32. doi: 10.1186/s13068-023-02283-6 (PMC9979520; doi:10.1186/s13068-023-02283-6)
Supplement: Supplementary file 4 — Additional file 4: Table S1. RT-PCR primer sets used in this study. [file 13068_2023_2283_MOESM4_ESM.docx]

**Table S1. RT-PCR primer sets used in this study.**

| Gene | Primer sequence (5’-3’) |
| --- | --- |
| *ppc* | F: CGCCTCCCCAAATTCTTCCT |
|  | R: CTGGCTGGTTCAAGAGCTGA |
| *sps* | F: CGCTAGGCGATAGAACTCCG |
|  | R: CGGCAGGTGTTCCAAGAGAT |
| *cscB* | F: GCGTTGCGTATCCTTTCC |
|  | R: GCACAATCCCAAGCGAACT |
| *glgA* | F: TCGCCCGTTCCTAAGACAAC |
|  | R: GGCTTTGTGGCTCGTCTAGT |
| *glgC* | F: TAGAATGAGGCGATCGTGCC |
|  | R: ACGTCTTCAAGCGGGATGTT |
| *glgP* | F: CGGATGATGTCTTGGAGCGA |
|  | R: GATGCGGTTGCCGACAAAAT |
