# Supplementary material for: Generation and comprehensive analysis of Synechococcus elongatus–Aspergillus nidulans co-culture system for polyketide production
Source: Biotechnol Biofuels Bioprod. 2023 Mar 1;16:32. doi: 10.1186/s13068-023-02283-6 (PMC9979520; doi:10.1186/s13068-023-02283-6)
Supplement: Supplementary file 5 — Additional file 5: Figure S4. Standard curve of neosartoricin B. [file 13068_2023_2283_MOESM5_ESM.docx]

**Figure S4.** **Standard curve of neosartoricin B.**
